# Supplementary material for: Genome architecture evolution in an invasive copepod species complex
Source: Nat Commun. 2025 Nov 21;16:10312. doi: 10.1038/s41467-025-65292-z (PMC12639140; doi:10.1038/s41467-025-65292-z)
Supplement: Supplementary file 1 — Supplementary Information [file 41467_2025_65292_MOESM1_ESM.pdf]

Supplementary Information

Genome architecture evolution in an invasive copepod species complex

Zhenyong Du[1,*], Johannes Wirtz[2], Yifei Joye Zhou[1], Anna Jenstead[1], Taylor Opgenorth[1], Angelise Puls[1], Cullan Meyer[1], Gregory W. Gelembiuk[1], Carol Eunmi Lee[1,*]

[1]Department of Integrative Biology, 430 Lincoln Drive, Birge Hall, University of Wisconsin, Madison, WI 53706, U.S.A.

[2]CEFE, Université de Montpellier, CNRS, EPHE, IRD, Montpellier 34090, France

*Corresponding Authors:

Zhenyong Du, zdu53@wisc.edu; Carol E. Lee, carollee@wisc.edu

Table of Contents:

Supplementary Figure 1. Phylogenetic relationships among clades (sibling species) of the *Eurytemora affinis* species complex.

Supplementary Figure 2. Karyotypes of three clades (sibling species) of the *Eurytemora affinis* complex based on fluorescence microscopy.

Supplementary Figure 3. Frequency plots of k-mer coverage for *E. gulfia* (Gulf clade) and *E. affinis* proper (Europe clade) of the *Eurytemora affinis* complex.

Supplementary Figure 4. Hi-C contact maps for three chromosomes of the *Eurytemora carolleeae* genome.

Supplementary Figure 5. Frequency distribution of the top 5% F_{ST} and θ_{π} outliers within random 1 Mb intervals on each chromosome of *Eurytemora carolleeae* (Atlantic clade of the *E. affinis* complex).

Supplementary Figure 6. Frequency distribution of the top 1% F_{ST} and θ_{π} outliers within random 1 Mb intervals on each chromosome of *Eurytemora carolleeae* (Atlantic clade of the *E. affinis* complex).

Supplementary Figure 7. Frequency distribution of the BayPass + 5% F_{ST} and θ_{π} outliers within random 1 Mb intervals on each chromosome of *Eurytemora carolleeae* (Atlantic clade of the *E. affinis* complex).

Supplementary Figure 8. Frequency distribution of the top 5% F_{ST} and θ_{π} outliers within random 2 Mb intervals on each chromosome of *Eurytemora carolleeae* (Atlantic clade of the *E. affinis* complex).

Supplementary Figure 9. Frequency distribution of the top 1% F_{ST} and θ_{π} outliers within random 2 Mb intervals on each chromosome of *Eurytemora carolleeae* (Atlantic clade of the *E. affinis* complex).

Supplementary Figure 10. Frequency distribution of the BayPass + 5% F_{ST} and θ_{π} outliers within random 2 Mb intervals on each chromosome of *Eurytemora carolleeae* (Atlantic clade of the *E. affinis* complex).

Supplementary Figure 11. Distribution of signatures of selection associated with salinity adaptation on seven chromosomes of *Eurytemora gulfia* (Gulf clade of the *E. affinis* complex).

Supplementary Figure 12. Frequency distribution of the top 5% F_{ST} and θ_{π} outliers within random 1 Mb intervals on each chromosome of *Eurytemora gulfia* (Gulf clade of the *E. affinis* complex).

Supplementary Figure 13. Frequency distribution of the top 1% F_{ST} and θ_{π} outliers within random 1 Mb intervals on each chromosome of *Eurytemora gulfia* (Gulf clade of the *E. affinis* complex).

Supplementary Figure 14. Frequency distribution of the BayPass + 5% F_{ST} and θ_{π} outliers within random 1 Mb intervals on each chromosome of *Eurytemora gulfia* (Gulf clade of the *E. affinis* complex).

Supplementary Figure 15. Frequency distribution of the top 5% F_{ST} and θ_{π} outliers within random 2 Mb intervals on each chromosome of *Eurytemora gulfia* (Gulf clade of the *E. affinis* complex).

Supplementary Figure 16. Frequency distribution of the top 1% F_{ST} and θ_{π} outliers within random 2 Mb intervals on each chromosome of *Eurytemora gulfia* (Gulf clade of the *E. affinis* complex).

Supplementary Figure 17. Frequency distribution of the BayPass + 5% F_{ST} and θ_{π} outliers within random 2 Mb intervals on each chromosome of *Eurytemora gulfia* (Gulf clade of the *E. affinis* complex).

Supplementary Figure 18. Patterns of linkage disequilibrium (LD) at the chromosomal fusion sites versus the genome-wide background.

Supplementary Figure 19. Demographic history of wild populations of *E. carolleeae* (Atlantic clade) and *E. gulfia* (Gulf clade) of the *Eurytemora affinis* complex.

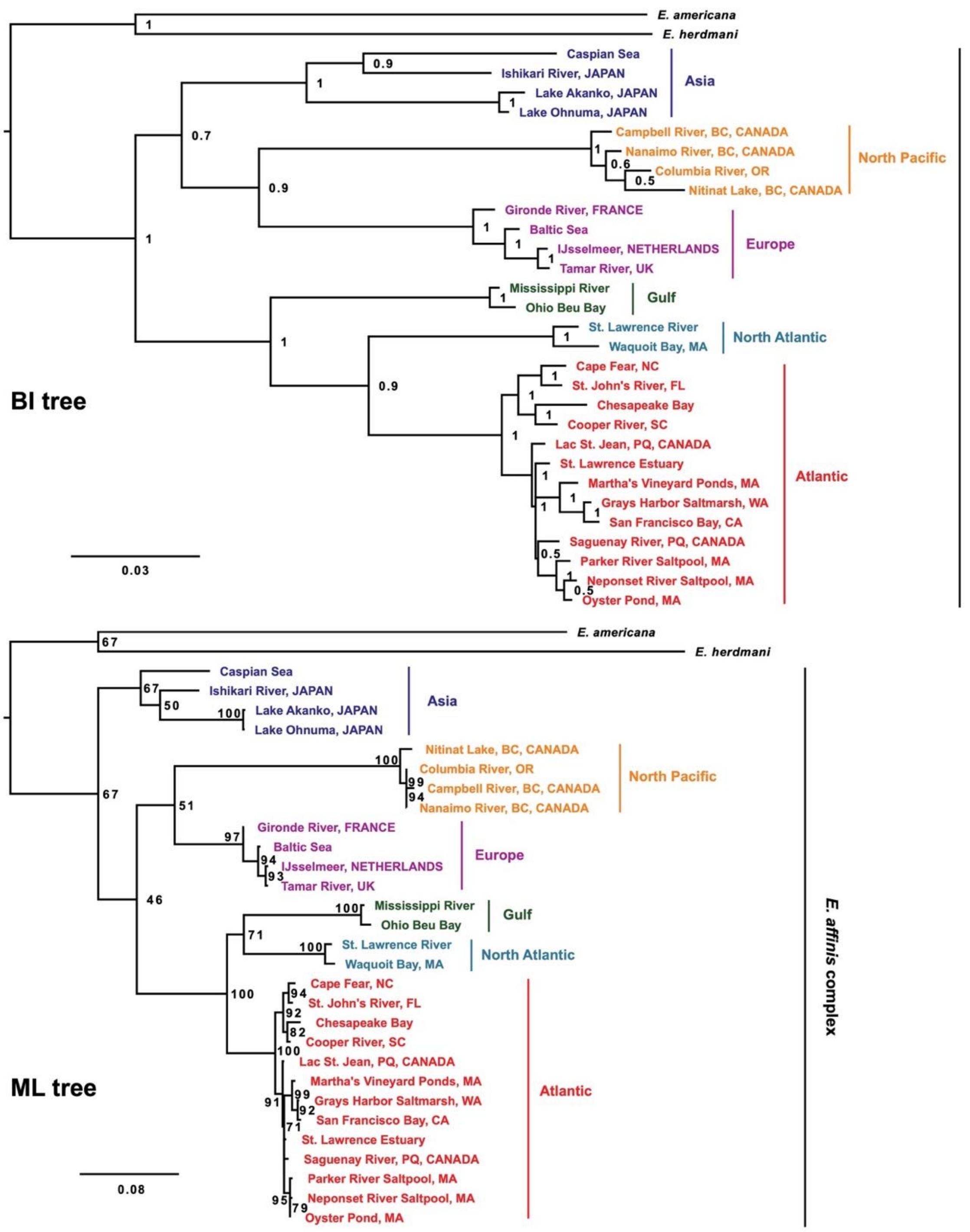

Supplementary Figure 1. Phylogenetic relationships among clades (sibling species) of the *Eurytemora affinis* species complex. These two phylogenies were constructed using concatenated cytochrome c oxidase I (*COI*) and *16S rRNA* gene sequences from Lee (2000)[1]. The upper phylogeny was constructed using Bayesian inference (BI) with MrBayes software[2]. The numbers at each node are the posterior probabilities. The lower phylogeny was generated using maximum-likelihood (ML) with IQ-Tree software[3]. The numbers at the nodes are bootstrap values for 1000 replicates.

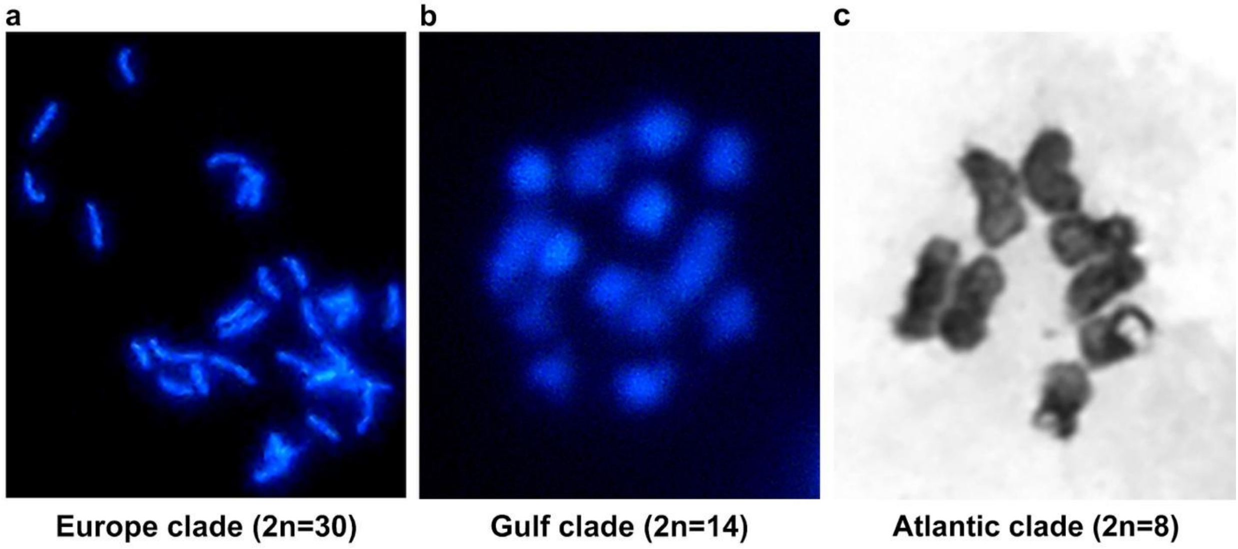

Supplementary Figure 2. Karyotypes of three clades (sibling species) of the *Eurytemora affinis* complex based on fluorescence microscopy. Karyotyping of embryos was performed to confirm chromosome counts. Embryos were dissected within 24 hours post-ovulation, treated with colchicine and a hypotonic solution, and fixed with Carnoy's fixative. Chromosomes were stained with DAPI and imaged using an Olympus BX60 epifluorescence microscope at the Newcomb Imaging Center, University of Wisconsin-Madison. The karyotype images show the chromosome counts for each clade: **(a)** 2n = 30 chromosomes are visible for the Europe clade (*E. affinis* proper), **(b)** 2n = 14 chromosomes for the Gulf clade (*E. gulfia*), and **(c)** 2n = 8 chromosomes for the Atlantic clade (*E. carolleeae*). The chromosome counts from the karyotype results are consistent with the Hi-C scaffolding results (Figure 2).

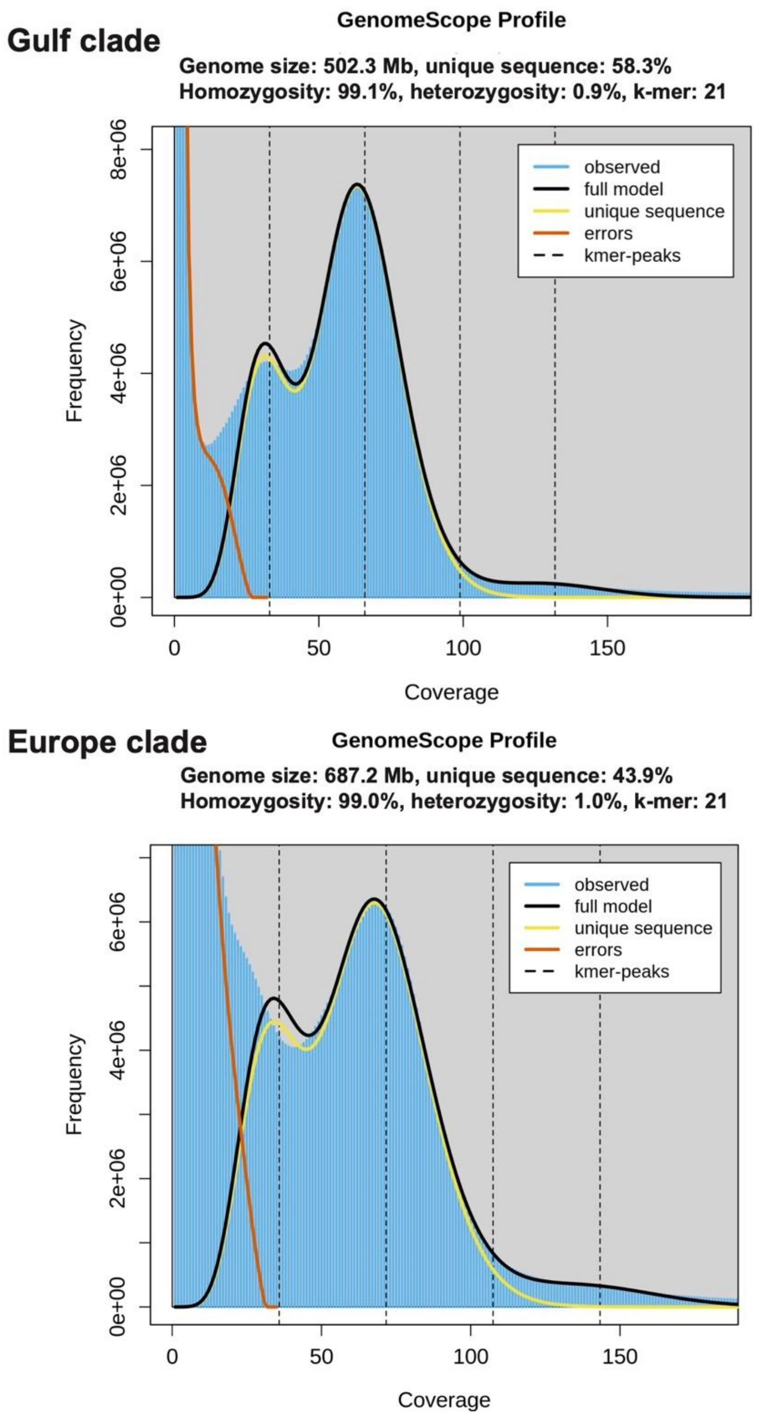

Supplementary Figure 3. Frequency plots of k-mer coverage for *E. gulfia* (Gulf clade) and *E. affinis* proper (Europe clade) of the *Eurytemora affinis* complex. The upper graph shows the k-mer coverage for *E. gulfia*, whereas the lower graph shows that for *E. affinis* proper. Estimates of genome size, proportion of non-repetitive unique sequences, homozygosity, and heterozygosity are shown above the plots. These results indicate that the *E. gulfia* genome has an approximate genome size of 502 Mb, with 0.9% heterozygosity and 42% repetitive sequence, whereas the *E. affinis* proper genome has an approximate genome size of 687 Mb, with 1.0% heterozygosity and 56% repetitive sequence.

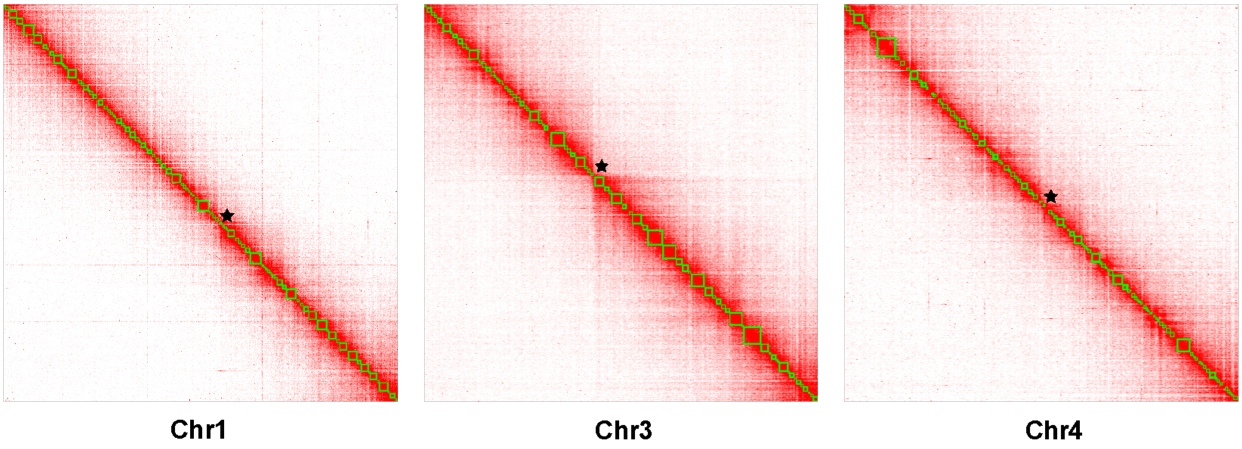

Supplementary Figure 4. Hi-C contact maps for three chromosomes of the *Eurytemora carolleeae* genome. The stars indicate the positions of the centromeres. The centromeres in the *E. gulfia* (Gulf clade) and *E. affinis* proper (Europe clade) genomes are not as distinct as those in the *E. carolleeae* (Atlantic clade) genome.

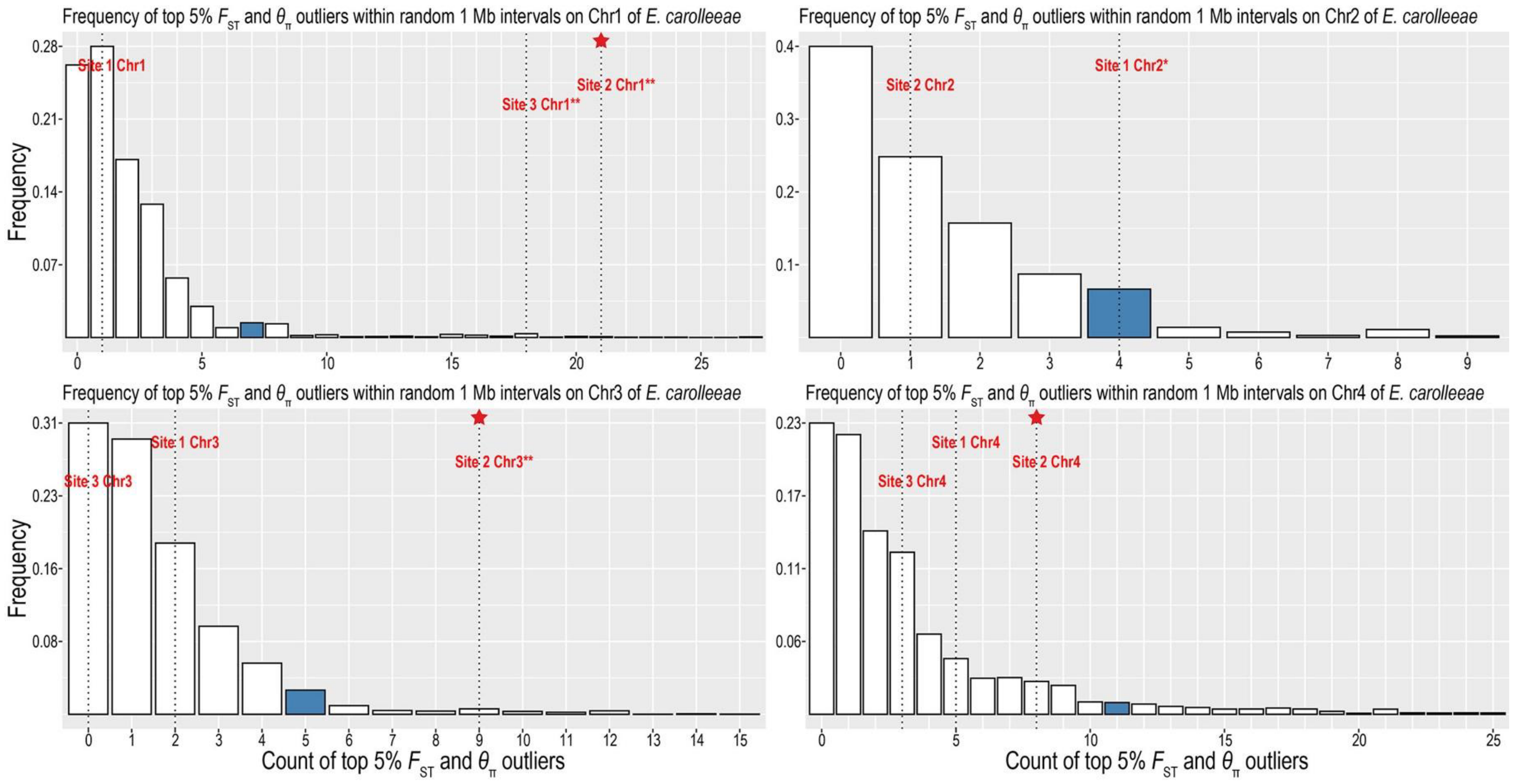

Supplementary Figure 5. Frequency distribution of the top 5% F_{ST} and θ_π outliers within random 1 Mb intervals on each chromosome of *Eurytemora carolleeae* (Atlantic clade of the *E. affinis* complex). Vertical dashed lines denote the fusion sites on the chromosomes. The numbering of the fusion sites on each chromosome is shown in Fig. 7. Asterisks denote significantly higher numbers of signatures of selection compared to the background distribution on the corresponding chromosomes. Detailed statistical results can be found in Supplementary Data 16. Significance levels are indicated as follows: *** for P-value < 0.001, ** for P-value < 0.01, and * for 0.01 < P-value < 0.05. Blue bars represent the position where the P-value equals 0.05. Centromeres are indicated by red stars. This figure corresponds to Supplementary Fig. 12 for the same analysis performed in *E. gulfia*.

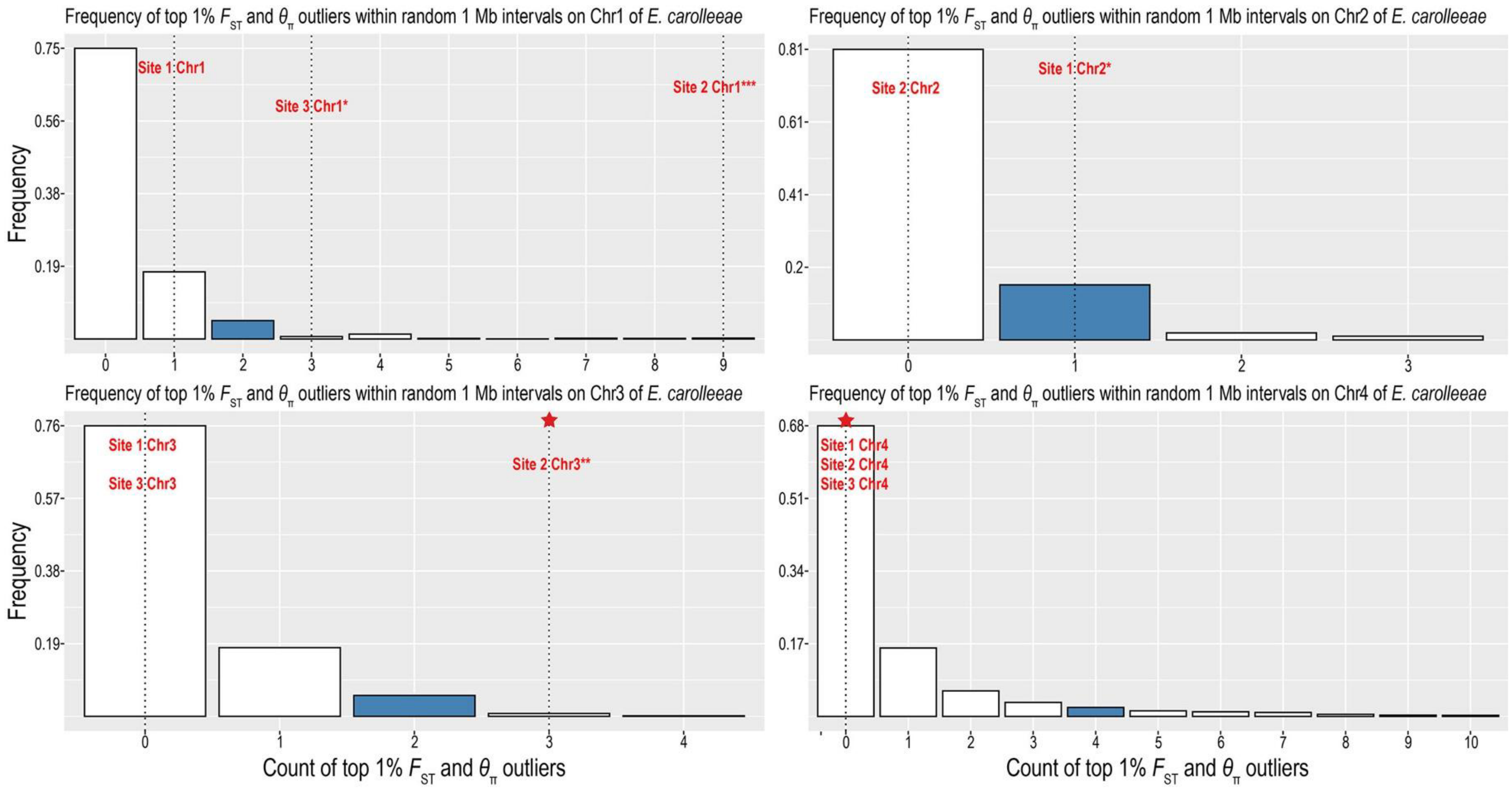

Supplementary Figure 6. Frequency distribution of the top 1% F_{ST} and θ_π outliers within random 1 Mb intervals on each chromosome of *Eurytemora carolleeae* (Atlantic clade of the *E. affinis* complex). Vertical dashed lines denote the fusion sites on the chromosomes. The numbering of the fusion sites on each chromosome is shown in Fig. 7. Asterisks denote significantly higher numbers of signatures of selection compared to the background distribution on the corresponding chromosomes. Detailed statistical results can be found in Supplementary Data 16. Significance levels are indicated as follows: *** for P-value < 0.001, ** for P-value < 0.01, and * for 0.01 < P-value < 0.05. Blue bars represent the position where the P-value equals 0.05. Centromeres are indicated by red stars. This figure corresponds to Supplementary Fig. 13 for the same analysis performed in *E. gulfia*.

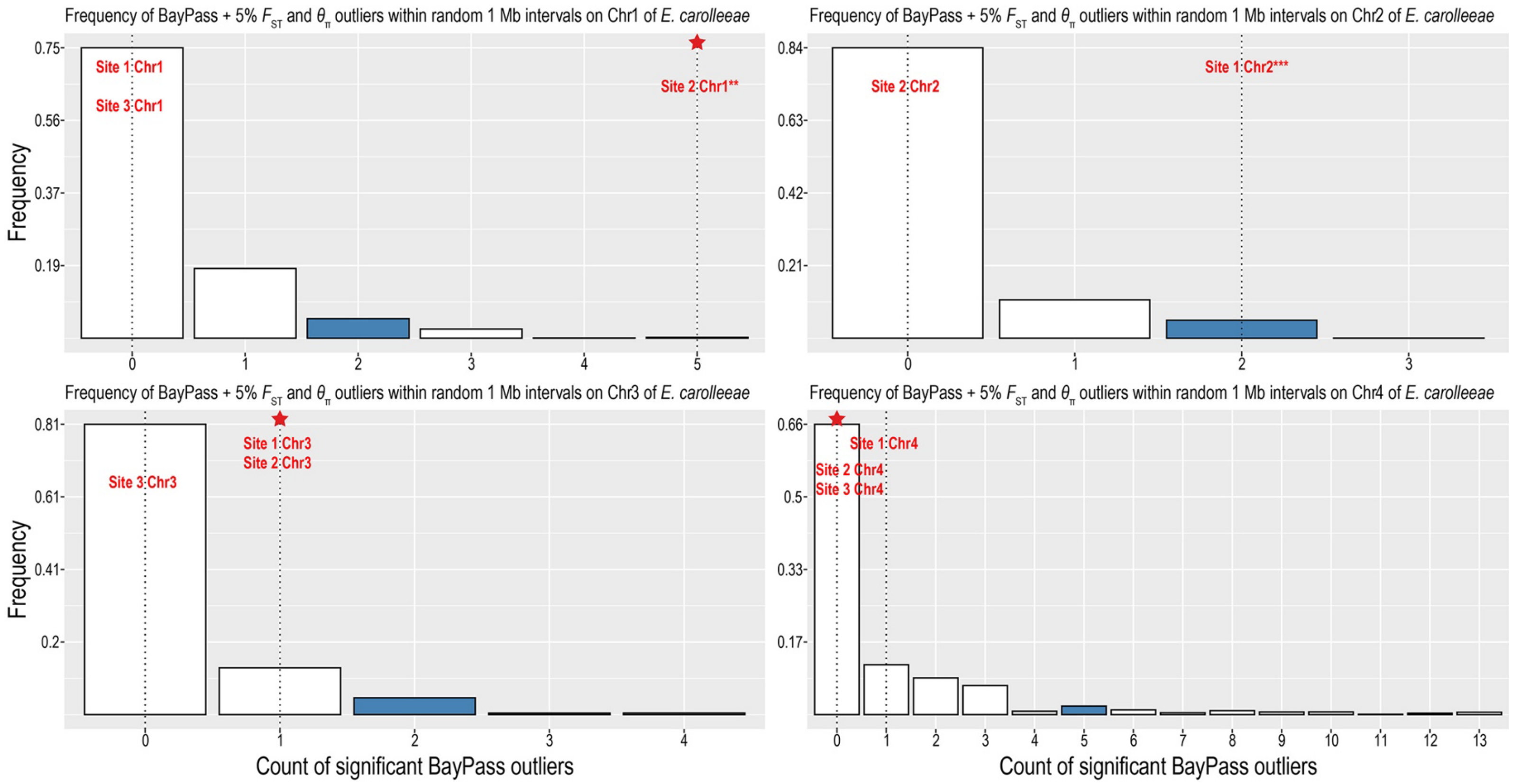

Supplementary Figure 7. Frequency distribution of the BayPass + 5% F_{ST} and θ_π outliers within random 1 Mb intervals on each chromosome of *Eurytemora carolleeae* (Atlantic clade of the *E. affinis* complex). Vertical dashed lines denote the fusion sites on the chromosomes. The numbering of the fusion sites on each chromosome is shown in Fig. 7. Asterisks denote significantly higher numbers of signatures of selection compared to the background distribution on the corresponding chromosomes. Detailed statistical results can be found in Supplementary Data 16. Significance levels are indicated as follows: *** for P-value < 0.001, ** for P-value < 0.01, and * for 0.01 < P-value < 0.05. Blue bars represent the position where the P-value equals 0.05. Centromeres are indicated by red stars. This figure corresponds to Supplementary Fig. 14 for the same analysis performed in *E. gulfia*.

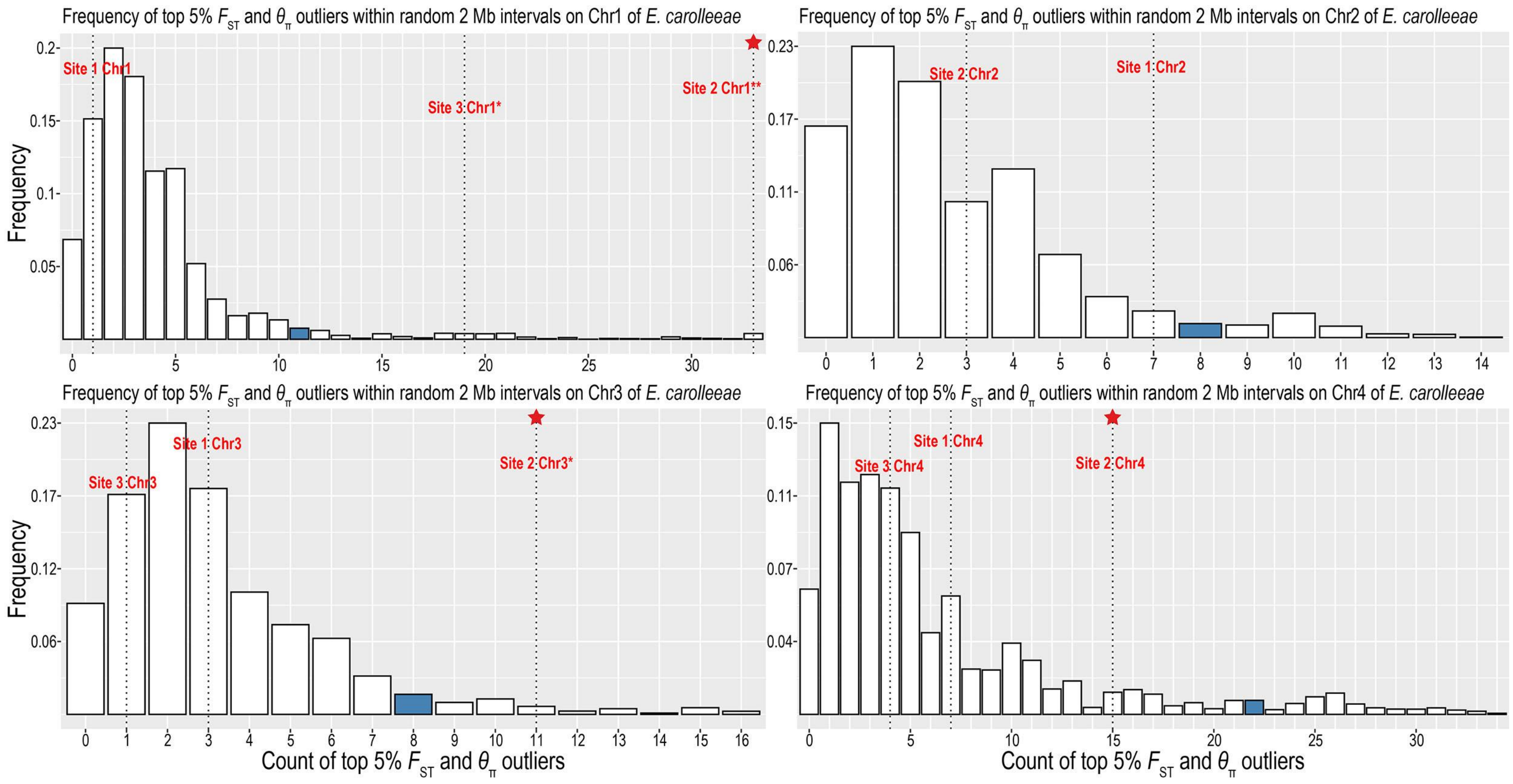

Supplementary Figure 8. Frequency distribution of the top 5% F_{ST} and θ_π outliers within random 2 Mb intervals on each chromosome of *Eurytemora carolleeae* (Atlantic clade of the *E. affinis* complex). Vertical dashed lines denote the fusion sites on the chromosomes. The numbering of the fusion sites on each chromosome is shown in Fig. 7. Asterisks denote significantly higher numbers of signatures of selection compared to the background distribution on the corresponding chromosomes. Detailed statistical results can be found in Supplementary Data 17. Significance levels are indicated as follows: *** for P-value < 0.001, ** for P-value < 0.01, and * for 0.01 < P-value < 0.05. Blue bars represent the position where the P-value equals 0.05. Centromeres are indicated by red stars. This figure corresponds to Supplementary Fig. 15 for the same analysis performed in *E. gulfia*.

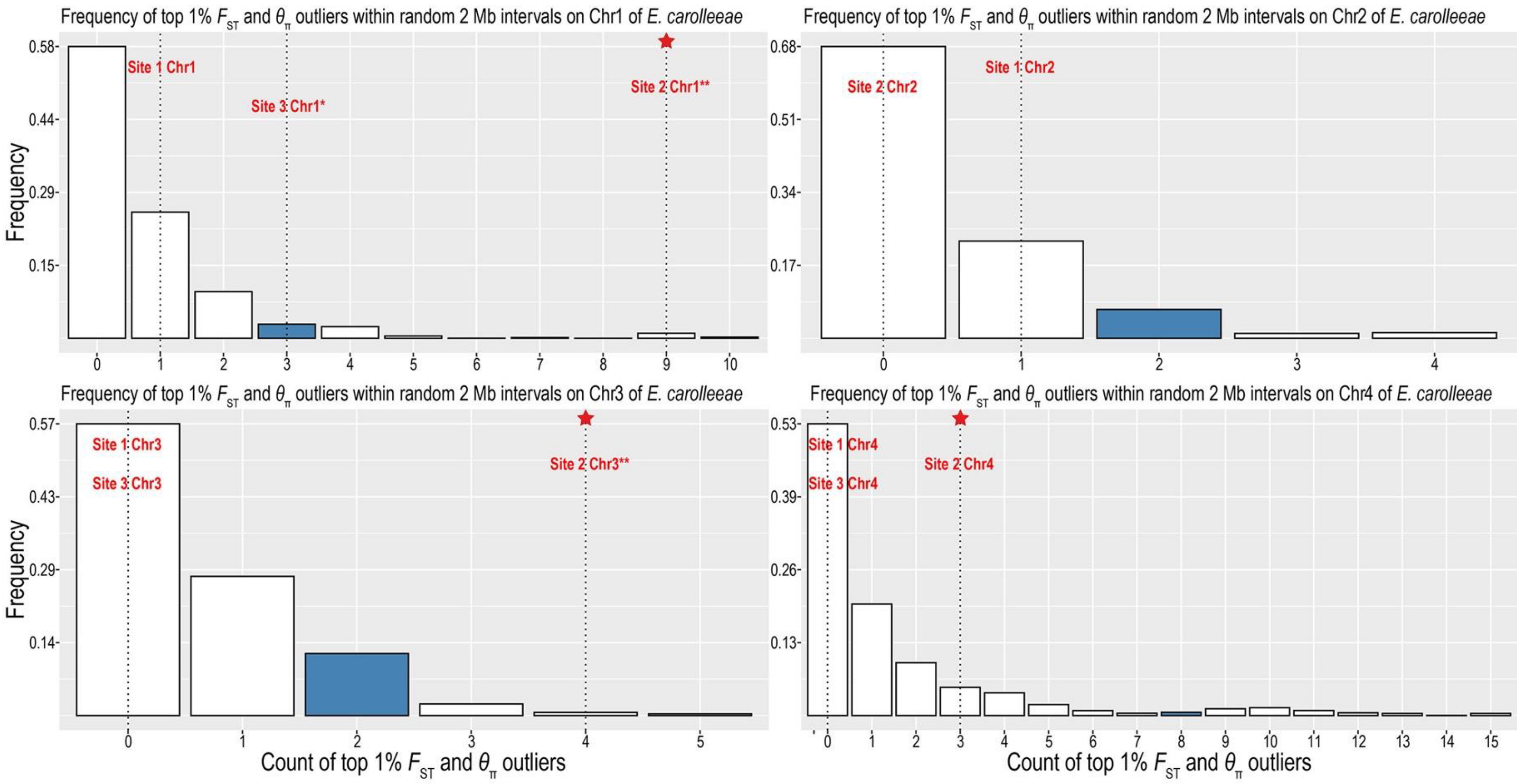

Supplementary Figure 9. Frequency distribution of the top 1% F_{ST} and θ_π outliers within random 2 Mb intervals on each chromosome of *Eurytemora carolleeae* (Atlantic clade of the *E. affinis* complex). Vertical dashed lines denote the fusion sites on the chromosomes. The numbering of the fusion sites on each chromosome is shown in Fig. 7. Asterisks denote significantly higher numbers of signatures of selection compared to the background distribution on the corresponding chromosomes. Detailed statistical results can be found in Supplementary Data 17. Significance levels are indicated as follows: *** for P-value < 0.001, ** for P-value < 0.01, and * for 0.01 < P-value < 0.05. Blue bars represent the position where the P-value equals 0.05. Centromeres are indicated by red stars. This figure corresponds to Supplementary Fig. 16 for the same analysis performed in *E. gulfia*.

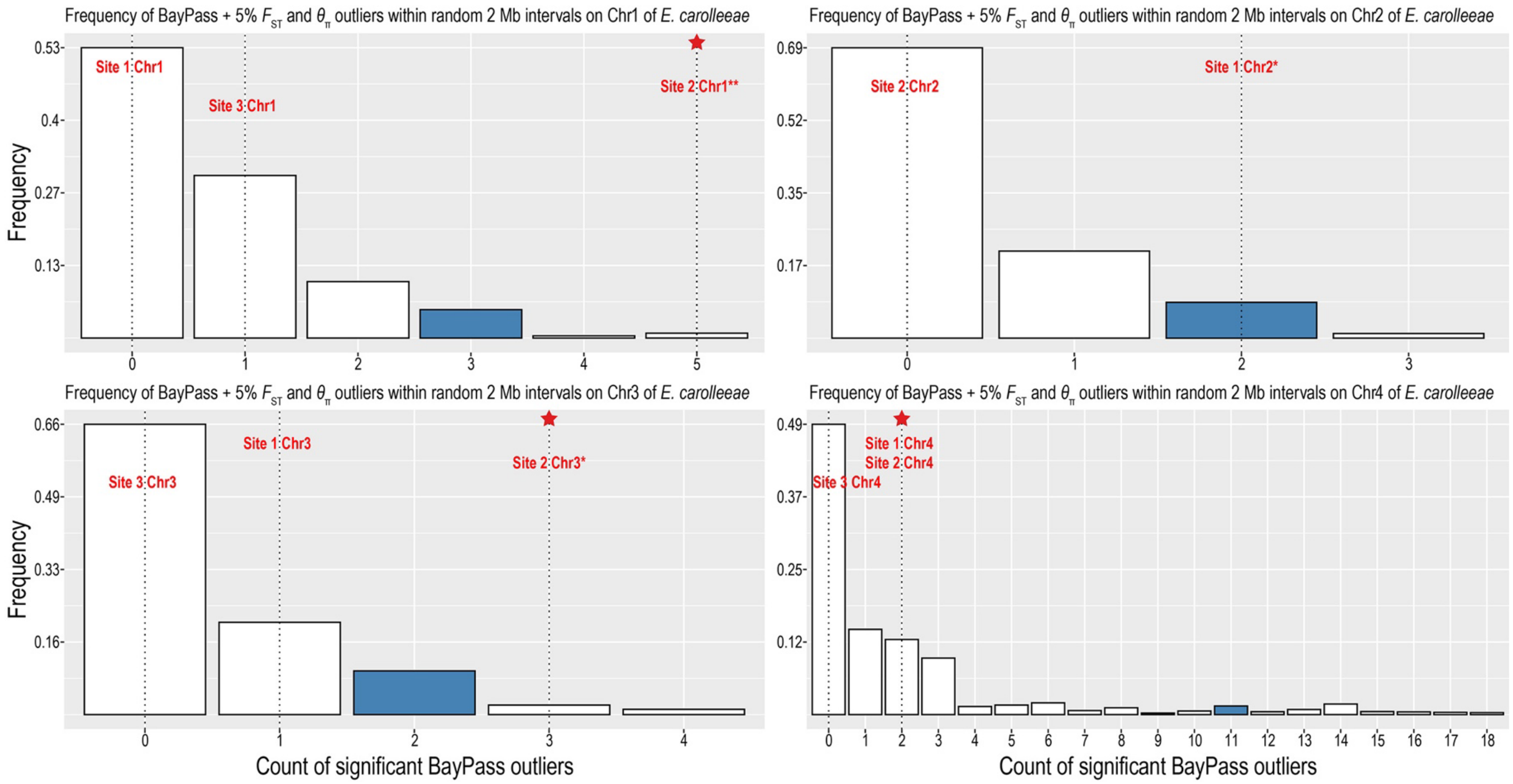

Supplementary Figure 10. Frequency distribution of the BayPass + 5% F_{ST} and θ_π outliers within random 2 Mb intervals on each chromosome of *Eurytemora carolleeae* (Atlantic clade of the *E. affinis* complex). Vertical dashed lines denote the fusion sites on the chromosomes. The numbering of the fusion sites on each chromosome is shown in Fig. 7. Asterisks denote significantly higher numbers of signatures of selection compared to the background distribution on the corresponding chromosomes. Detailed statistical results can be found in Supplementary Data 17. Significance levels are indicated as follows: *** for *P*-value < 0.001, ** for *P*-value < 0.01, and * for 0.01 < *P*-value < 0.05. Centromeres are indicated by red arrows. Blue bars represent the position where the *P*-value equals 0.05. Centromeres are indicated by red stars. This figure corresponds to Supplementary Fig. 17 for the same analysis performed in *E. gulfia*.

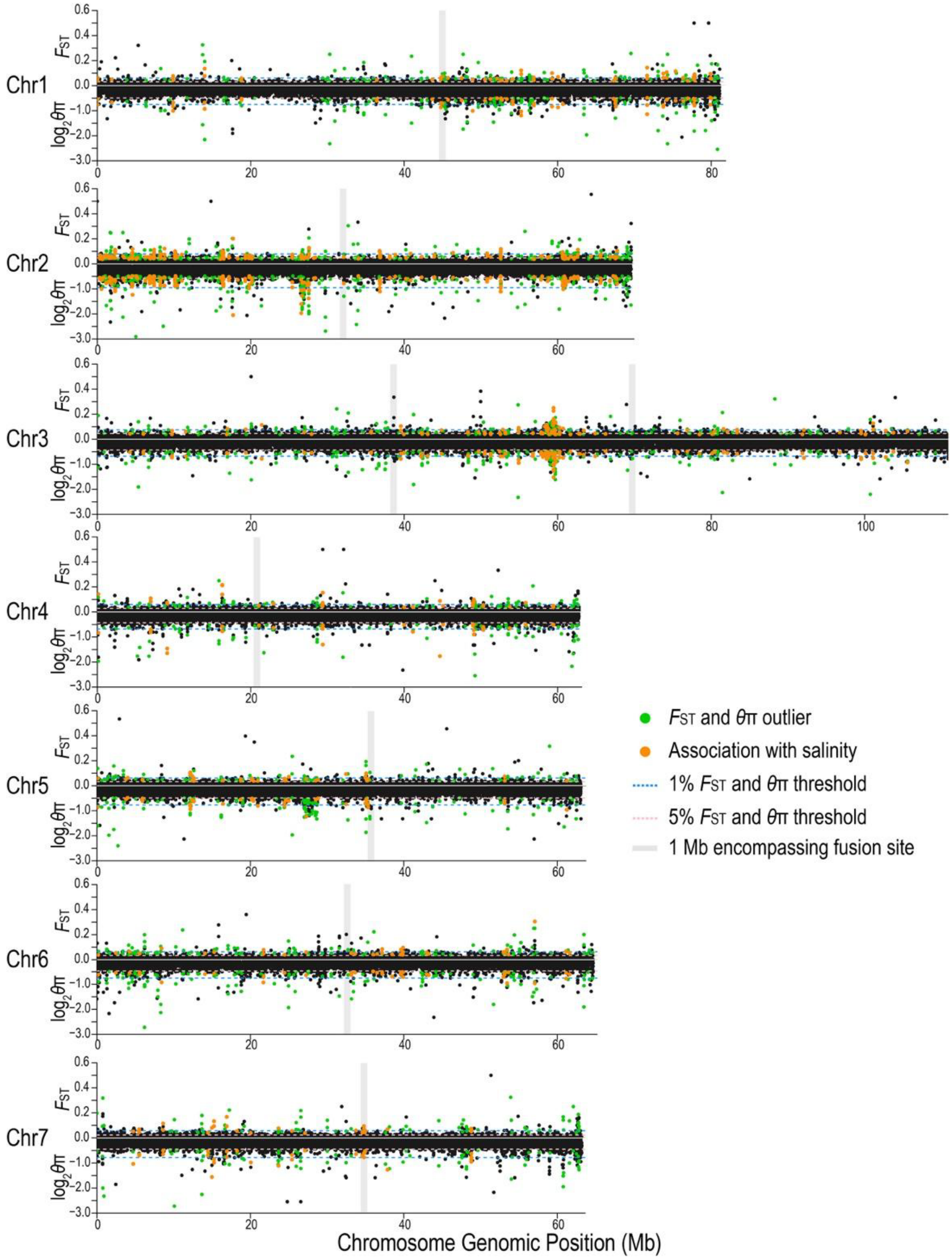

Supplementary Figure 11. Distribution of signatures of selection associated with salinity adaptation on seven chromosomes of *Eurytemora gulfia* (Gulf clade of the *E. affinis* complex). Each panel displays the distribution of selection signatures on a chromosome. The upper Manhattan plots (above 0) represent selection signatures detected by F_{ST}, with the y-axis showing F_{ST} values. The lower Manhattan plots (below 0) display signatures of selection detected by nucleotide diversity, expressed as the θ_{π} ratio ($\theta_{\pi\text{-invasive}}/\theta_{\pi\text{-native}}$), with the y-axis showing $\log_2(\theta_{\pi\text{-invasive}}/\theta_{\pi\text{-native}})$ values. Green dots highlight outliers shared between the top 5% F_{ST} and the lowest 5% $\log_2(\theta_{\pi\text{-invasive}}/\theta_{\pi\text{-native}})$. Orange dots indicate these 5% outliers (green dots) significantly associated with salinity, as identified by BayPass. Horizontal pink and blue dashed lines denote the thresholds for the top 5% and 1% values, respectively. Vertical gray strips mark the 1 Mb regions encompassing the fusion sites on each chromosome. This figure corresponds to Figs. 7a–d for the chromosome plots of *E. carolleeae*. Source data are provided as a Source Data file.

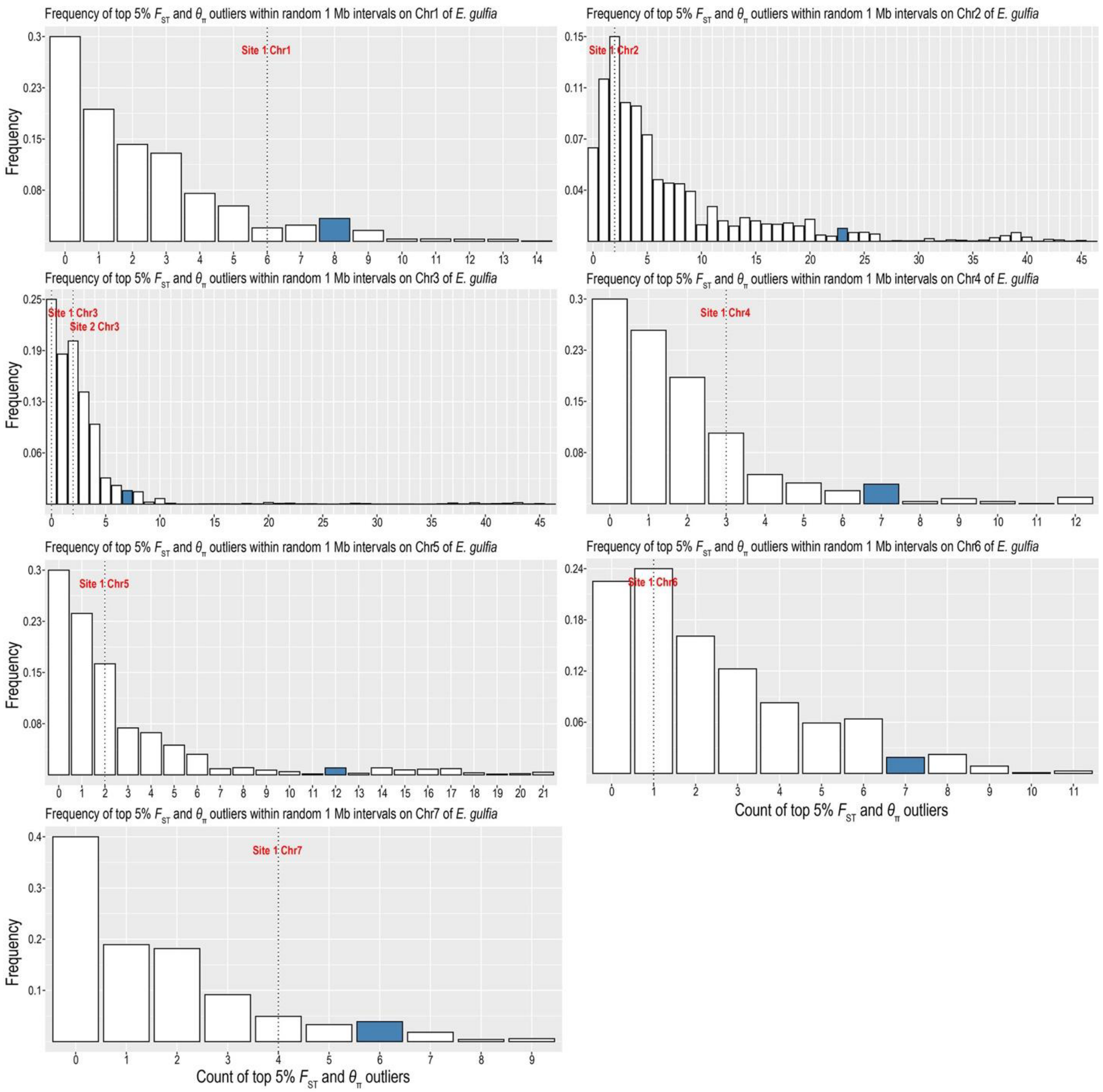

Supplementary Figure 12. Frequency distribution of the top 5% F_{ST} and θ_π outliers within random 1 Mb intervals on each chromosome of *Eurytemora gulfia* (Gulf clade of the *E. affinis* complex). Vertical dashed lines denote the fusion sites on the chromosomes. The positions of the fusion sites on each chromosome is shown in Supplementary Fig. 11. Detailed statistical results can be found in Supplementary Data 18. None of these fusion sites show significantly higher numbers of signatures of selection compared to the background distribution on the corresponding chromosomes. Blue bars represent the position where the *P*-value equals 0.05. This figure corresponds to Supplementary Fig. 5 for the same analysis performed in *E. carolleeae*.

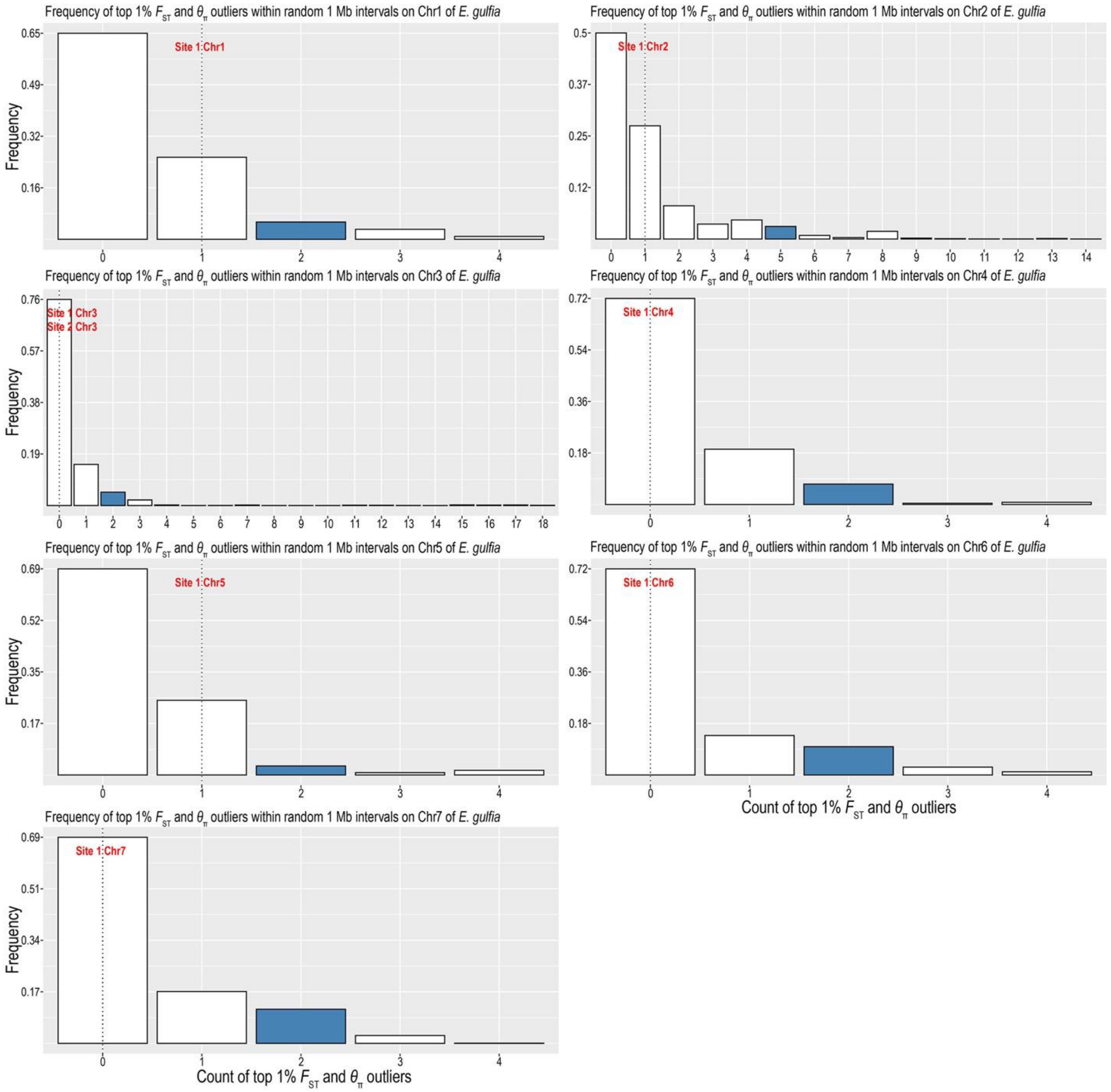

Supplementary Figure 13. Frequency distribution of the top 1% F_{ST} and θ_π outliers within random 1 Mb intervals on each chromosome of *Eurytemora gulfia* (Gulf clade of the *E. affinis* complex). Vertical dashed lines denote the fusion sites on the chromosomes. The positions of the fusion sites on each chromosome is shown in Supplementary Fig. 11. Detailed statistical results can be found in Supplementary Data 18. None of these fusion sites denote significantly higher numbers of signatures of selection compared to the background distribution on the corresponding chromosomes. Blue bars represent the position where the *P*-value equals 0.05. This figure corresponds to Supplementary Fig. 6 for the same analysis performed in *E. carolleeae*.

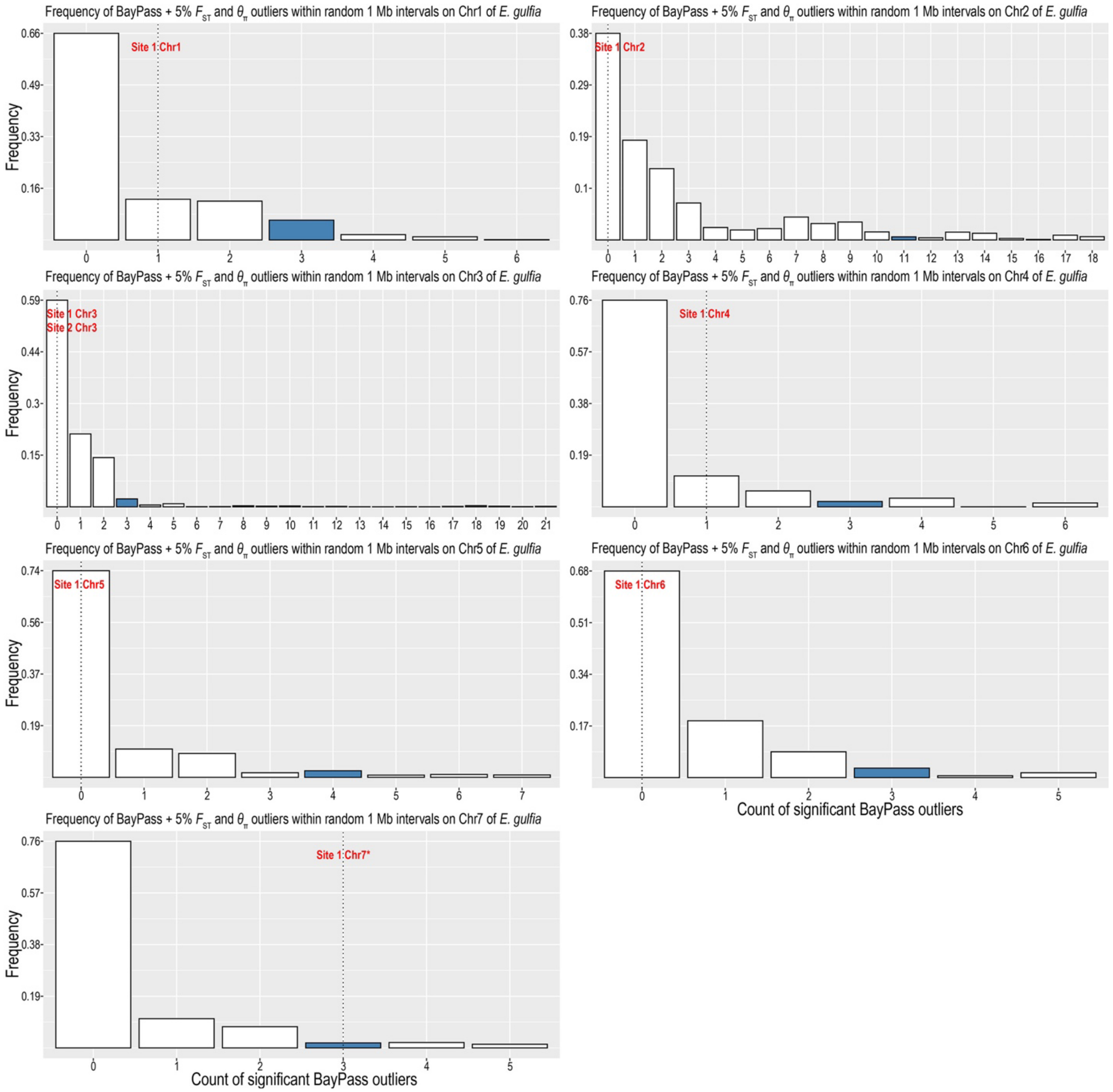

Supplementary Figure 14. Frequency distribution of the BayPass + 5% F_{ST} and θ_π outliers within random 1 Mb intervals on each chromosome of *Eurytemora gulfia* (Gulf clade of the *E. affinis* complex). Vertical dashed lines denote the fusion sites on the chromosomes. The positions of the fusion sites on each chromosome is shown in Supplementary Fig. 11. Detailed statistical results can be found in Supplementary Data 18. Significance levels are indicated by * for *P*-values less than 0.05. The fusion site on Chr7 shows significantly higher numbers of signatures of selection compared to the background distribution on the corresponding chromosomes. Blue bars represent the position where the *P*-value equals 0.05. This figure corresponds to Supplementary Fig. 7 for the same analysis performed in *E. carolleeae*.

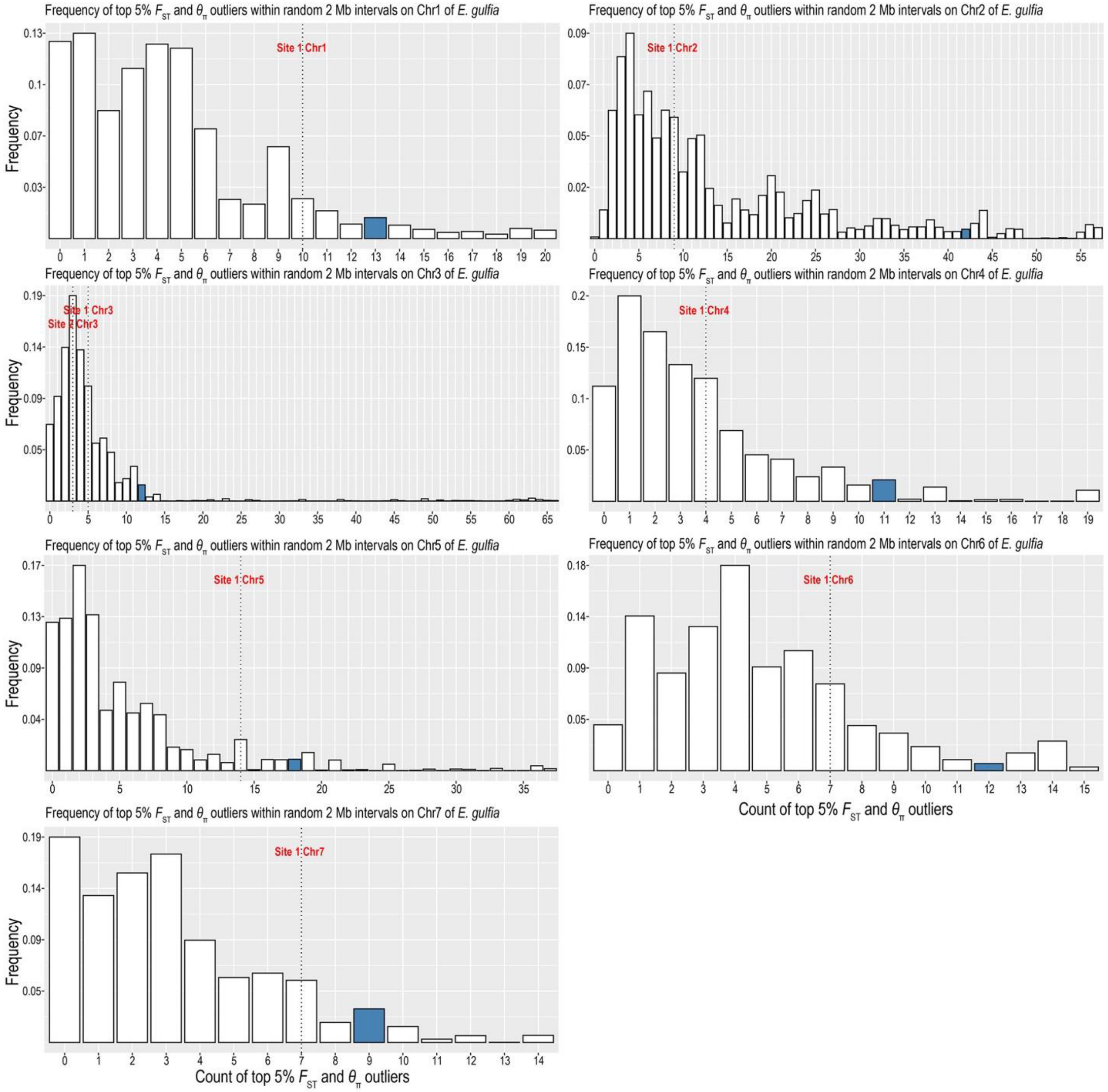

Supplementary Figure 15. Frequency distribution of the top 5% F_{ST} and θ_π outliers within random 2 Mb intervals on each chromosome of *Eurytemora gulfia* (Gulf clade of the *E. affinis* complex). Vertical dashed lines denote the fusion sites on the chromosomes. The positions of the fusion sites on each chromosome is shown in Supplementary Fig. 11. Detailed statistical results can be found in Supplementary Data 19. None of these fusion sites show significantly higher numbers of signatures of selection compared to the background distribution on the corresponding chromosomes. Blue bars represent the position where the *P*-value equals 0.05. This figure corresponds to Supplementary Fig. 8 for the same analysis performed in *E. carolleeae*.

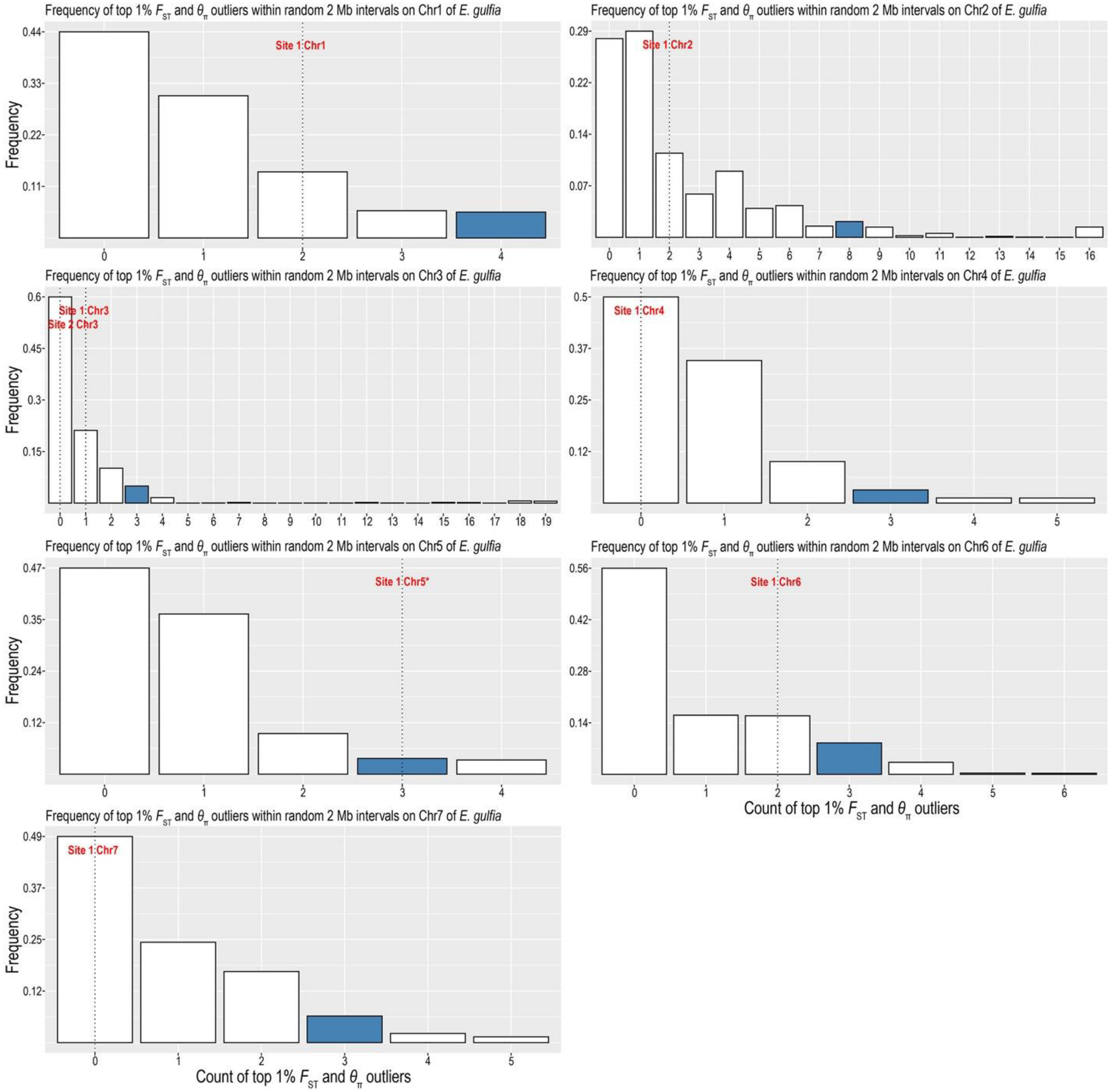

Supplementary Figure 16. Frequency distribution of the top 1% F_{ST} and θ_π outliers within random 2 Mb intervals on each chromosome of *Eurytemora gulfia* (Gulf clade of the *E. affinis* complex). Vertical dashed lines denote the fusion sites on the chromosomes. The positions of the fusion sites on each chromosome is shown in Supplementary Fig. 11. Detailed statistical results can be found in Supplementary Data 19. Significance levels are indicated by * for *P*-values less than 0.05. The fusion site on Chr5 shows significantly higher numbers of signatures of selection compared to the background distribution on the corresponding chromosomes. Blue bars represent the position where the *P*-value equals 0.05. This figure corresponds to Supplementary Fig. 9 for the same analysis performed in *E. carolleeae*.

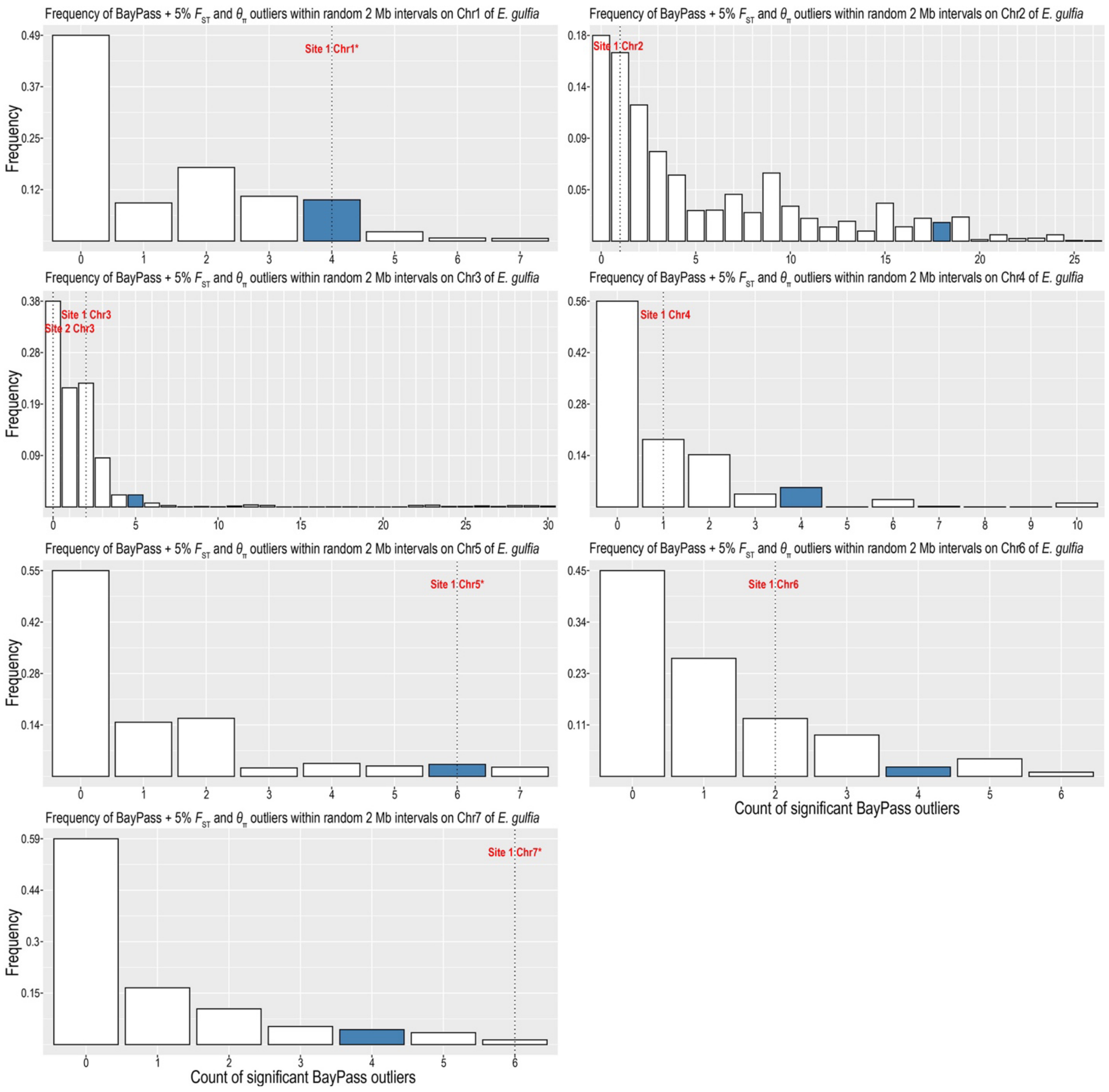

Supplementary Figure 17. Frequency distribution of the BayPass + 5% F_{ST} and θ_π outliers within random 2 Mb intervals on each chromosome of *Eurytemora gulfia* (Gulf clade of the *E. affinis* complex). Vertical dashed lines denote the fusion sites on the chromosomes. The positions of the fusion sites on each chromosome is shown in Supplementary Fig. 11. Detailed statistical results can be found in Supplementary Data 19. Significance levels are indicated by * for *P*-values less than 0.05. The fusion sites on Chr1, 5, and 7 show significantly higher numbers of signatures of selection compared to the background distribution on the corresponding chromosomes. Blue bars represent the position where the *P*-value equals 0.05. This figure corresponds to Supplementary Fig. 10 for the same analysis performed in *E. carolleeae*.

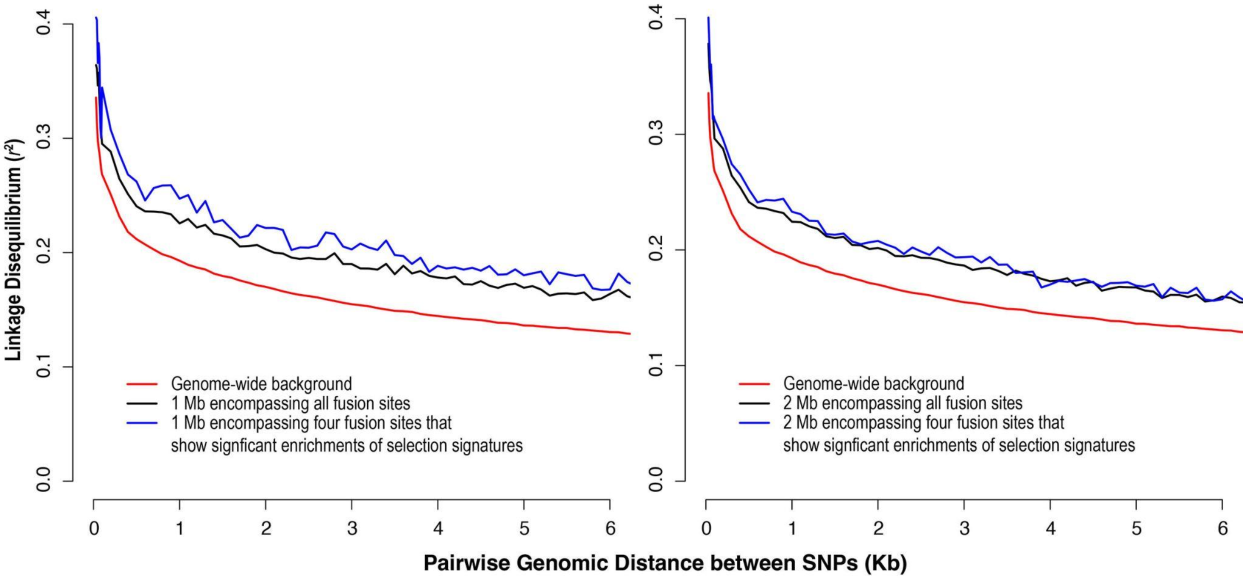

Supplementary Figure 18. Patterns of linkage disequilibrium (LD) at the chromosomal fusion sites versus the genome-wide background. LD decay was analyzed between 1 Mb (left) and 2 Mb (right) genomic regions encompassing chromosomal fusion sites versus genome-wide background regions. Analyses were based on whole-genome sequencing of 14 *Eurytemora carolleeae* individuals collected from Baie de L'Isle Verte, St. Lawrence Estuary, Quebec in 2022. Pairwise LD (r^2) was calculated using PopLDdecay[4]. Source data are provided as a Source Data file.

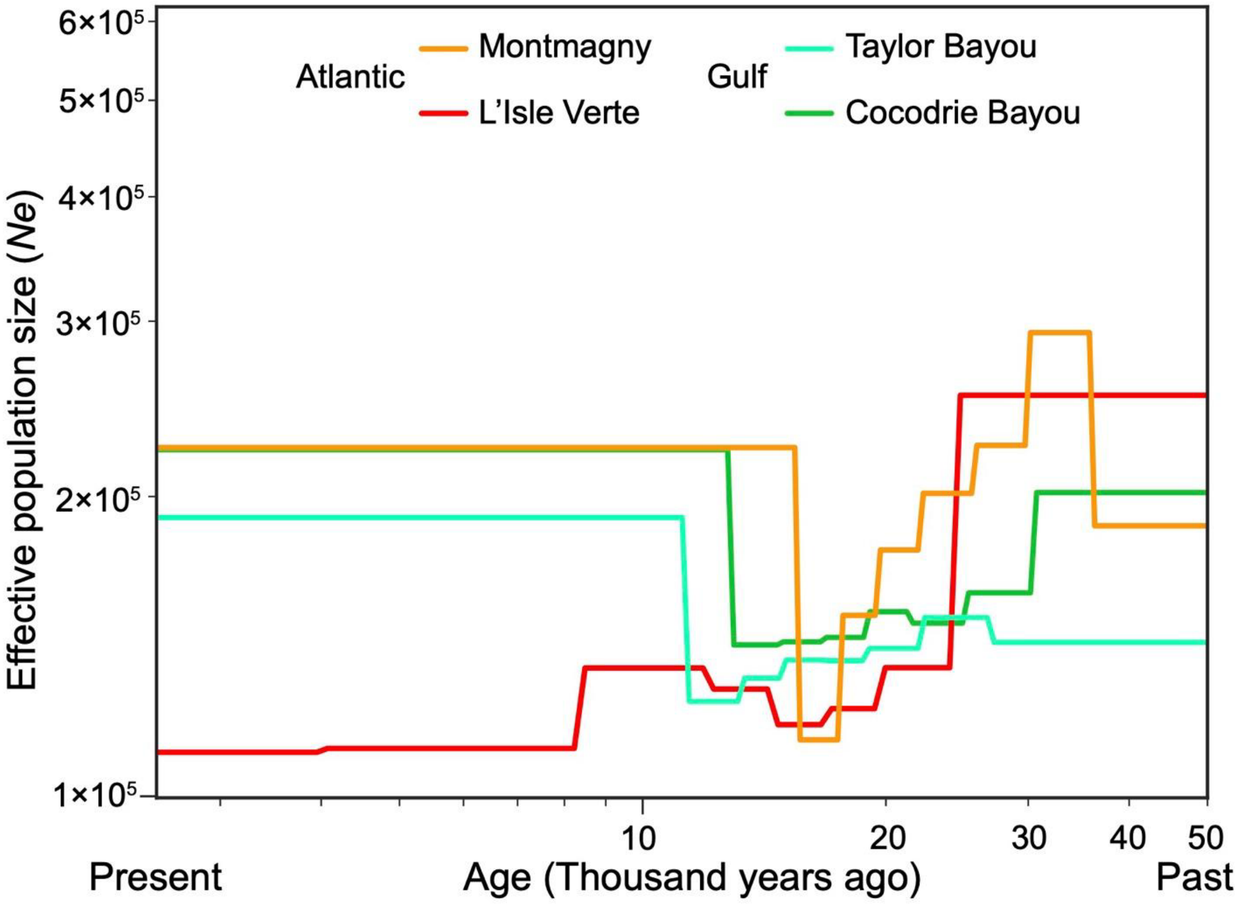

Supplementary Figure 19. Demographic history of wild populations of *E. carolleeae* (Atlantic clade) and *E. gulfia* (Gulf clade) of the *Eurytemora affinis* complex. The trajectories of the colored lines indicate changes in effective population size (N_e) over time inferred by SMC++[5]. This analysis suggests a decrease of N_e during the Last Glacial Maximum (18–26 thousand years ago) in both the Atlantic and Gulf clades. Locations of these populations are shown in Stern and Lee (2020)[6] and Supplementary Data 23.

References

1 Lee CE. Global phylogeography of a cryptic copepod species complex and reproductive isolation between genetically proximate "populations". *Evolution* **54**, 2014-2027 (2000).

2 Ronquist F, Huelsenbeck JP. MrBayes 3: Bayesian phylogenetic inference under mixed models. *Bioinformatics* **19**, 1572-1574 (2003).

3 Trifinopoulos J, Nguyen LT, von Haeseler A, Minh BQ. W-IQ-TREE: a fast online phylogenetic tool for maximum likelihood analysis. *Nucleic Acids Res* **44**, W232-W235 (2016).

4 Zhang C, Dong SS, Xu JY, He WM, Yang TL. PopLDdecay: a fast and effective tool for linkage disequilibrium decay analysis based on variant call format files. *Bioinformatics* **35**, 1786-1788 (2019).

5 Terhorst J, Kamm JA, Song YS. Robust and scalable inference of population history from hundreds of unphased whole genomes. *Nat Genet* **49**, 303-309 (2017).

6 Stern DB, Lee CE. Evolutionary origins of genomic adaptations in an invasive copepod. *Nat Ecol Evol* **4**, 1084-1094 (2020).
