# Supplementary material for: Genome architecture evolution in an invasive copepod species complex
Source: Nat Commun. 2025 Nov 21;16:10312. doi: 10.1038/s41467-025-65292-z (PMC12639140; doi:10.1038/s41467-025-65292-z)
Supplement: Supplementary file 2 — Description of Additional Supplementary Files [file 41467_2025_65292_MOESM2_ESM.pdf]

Description of Additional Supplementary Files

File Name: Supplementary Data 1
Description: Description of sequence data of three sibling species from the *Eurytemora affinis* complex obtained from previous and current studies.

File Name: Supplementary Data 2
Description: Genome assembly statistics of three reference genomes of the *Eurytemora affinis* complex.

File Name: Supplementary Data 3
Description: Repetitive sequences in the *Eurytemora gulfia* genome.

File Name: Supplementary Data 4
Description: Repetitive sequences in the *Eurytemora affinis* proper genome.

File Name: Supplementary Data 5
Description: Functional annotation of protein-coding genes in the *Eurytemora gulfia* and *E. affinis* proper genomes.

File Name: Supplementary Data 6
Description: Copepod and daphnid genome assemblies used in the comparative genomic analyses.

File Name: Supplementary Data 7
Description: GO enrichment of expanded genes in the *Eurytemora affinis* complex.

File Name: Supplementary Data 8
Description: GO enrichment of contracted genes in the *Eurytemora affinis* complex.

File Name: Supplementary Data 9
Description: GO enrichment of unique genes in the *Eurytemora affinis* complex.

File Name: Supplementary Data 10
Description: GO enrichment of lost genes in the *Eurytemora affinis* complex.

File Name: Supplementary Data 11
Description: Key ion transporter gene positions relative to the edges of each chromosome in the three sibling species of the *Eurytemora affinis* complex.

File Name: Supplementary Data 12
Description: Genes under selection associated with salinity adaptation in *Eurytemora carolleeae* with functional annotations and detection methods.

File Name: Supplementary Data 13
Description: GO enrichment of genes under selection associated with salinity

adaptation in *Eurytemora carolleeae*.

File Name: Supplementary Data 14
Description: Genes under selection associated with salinity adaptation in *Eurytemora gulfia* with functional annotations and detection methods.

File Name: Supplementary Data 15
Description: GO enrichment of genes under selection associated with salinity adaptation in *Eurytemora gulfia*.

File Name: Supplementary Data 16
Description: Statistical analyses on selection signatures associated with salinity adaptation within random 1 Mb intervals on each chromosome of *Eurytemora carolleeae*.

File Name: Supplementary Data 17
Description: Statistical analyses on selection signatures associated with salinity adaptation within random 2 Mb intervals on each chromosome of *Eurytemora carolleeae*.

File Name: Supplementary Data 18
Description: Statistical analyses on selection signatures associated with salinity adaptation within random 1 Mb intervals on each chromosome of *Eurytemora gulfia*.

File Name: Supplementary Data 19
Description: Statistical analyses on selection signatures associated with salinity adaptation within random 2 Mb intervals on each chromosome of *Eurytemora gulfia*.

File Name: Supplementary Data 20
Description: GC content of telomere regions and fusion sites of each chromosome in *Eurytemora carolleeae* and *E. gulfia*.

File Name: Supplementary Data 21
Description: Morphological data of female copepod samples used for the PCA analysis of the *Eurytemora affinis* complex.

File Name: Supplementary Data 22
Description: Morphological data of male copepod samples used for the PCA analysis of the *Eurytemora affinis* complex.

File Name: Supplementary Data 23
Description: Sample information used in the population genomic analyses of the *Eurytemora affinis* complex.
